# Violence against People with Disability in England and Wales: Findings from a National Cross-Sectional Survey

**DOI:** 10.1371/journal.pone.0055952

**Published:** 2013-02-20

**Authors:** Hind Khalifeh, Louise M. Howard, David Osborn, Paul Moran, Sonia Johnson

**Affiliations:** 1 Mental Health Sciences Unit, University College London (UCL), London, England; 2 Institute of Psychiatry, King’s College London, London, England; Cardiff University, United Kingdom

## Abstract

**Background:**

The recent World Report on Disability highlighted violence as a leading cause of morbidity among disabled people. However, we know little about the extent to which people with disability experience different violence types, and associated health/economic costs. The recent introduction of disability measures into the England&Wales victimization survey provided an opportunity to address this gap.

**Methods and Findings:**

Analysis of the 2009/10 British Crime Survey (BCS), a nationally representative cross-sectional survey of 44,398 adults living in residential households in England&Wales. Using multivariate logistic regression, we estimated the relative odds of being a victim of past-year violence (physical/sexual domestic or non-domestic violence) in people with disability compared to those without, after adjusting for socio-demographics, behavioural and area confounders. 1256/44398(2.4%) participants had one or more disabilities including mental illness (‘mental illness’) and 7781(13.9%) had one or more disabilities excluding mental illness (‘non-mental disability’). Compared with the non-disabled, those with mental illness had adjusted relative odds (aOR) of 3.0(95% confidence interval (CI) 2.3–3.8) and those with non-mental disability had aOR of 1.8(95% CI: 1.5–2.2) of being a victim of past-year violence (with similar relative odds for domestic and non-domestic violence). Disabled victims were more likely to suffer mental ill health as a result of violence than non-disabled victims. The proportion of violence that could be attributed to the independent effect of disability in the general population was 7.5%(CI 5.7–9.3%), at an estimated cost of £1.51 billion. The main study limitation is the exclusion of institutionalised people with disability.

**Conclusions:**

People with disability are at increased risk of being victims of domestic and non-domestic violence, and of suffering mental ill health when victimized. The related public health and economic burden calls for an urgent assessment of the causes of this violence, and national policies on violence prevention in this vulnerable group.

## Introduction

There are more than a billion people with physical or mental disability worldwide, comprising around 15% of the global population. [1] The recent World Report on Disability [1] highlights physical and sexual violence against people with disability as a major risk factor for ill health in this group. In the non-disabled population, violence contributes significantly to the global burden of injuries, physical and mental health problems, substance misuse and death. [2] The health impact of violence among the disabled is likely to be compounded by pre-existing morbidity and difficult social circumstances. The 2006 United Nations Convention on the Rights of Persons with Disabilities highlights the entitlement of this vulnerable group to ‘freedom from exploitation, violence and abuse’ and obliges member states to ‘take all appropriate measures’ to prevent violence and rehabilitate victims. In order to meet these obligations, we need to better understand the epidemiology of violence in this population.

Recent reports by the Equality and Human Rights Commission found evidence for high levels of violence against people with disability, but called for “definitive data…on the scale, severity and nature of disability harassment”. [3], [4] A recent systematic review found that past-year violence was experienced by 24% of people with mental illness and 3% of people with non-specific impairment (with pooled adjusted odds ratios of 3.9 and 1.5 respectively compared with the non-disabled), but highlighted important gaps in the evidence base. [5] We identified three key unanswered questions of relevance to policy makers, which we address in this study. Firstly, we do not know who among the disabled is most at risk, and what type of violence they are most likely to suffer from [5]. Past evidence suggested that those with mental illness were at particularly high risk, but this was largely based on comparing clinical samples of people with severe mental illness to general population samples of people with self-defined physical disability. Secondly, there is little evidence on the health impact of violence in this group, which may be magnified by chronic illness and poor psychosocial resources. [6] Finally, we do not know what proportion of violence in the population as a whole (and in those with disability) is explained by disability-associated risk. Policy makers need answers to these questions in order to design and target cost-effective interventions.

In the UK, there are 10 million people living with a limiting disability. [7] The government recently recommended the addition of disability measures to major national surveys, in order to estimate unmet needs in this population. The British Crime Survey (BCS), a large national victimization survey, introduced a measure of disability subtypes for the first time in 2009. This provided a timely opportunity to address the following hypotheses/questions: (1) Are people with disability at greater risk of violence (and violence subtypes) than those without disability, and do people with mental illness have a greater risk than those with physical/other disabilities? (2) Do disabled victims experience more severe health problems following violence than non-disabled victims? (3) What proportion of violence victimisation in the population as a whole (and in those with disability) is attributable to the independent effect of disability (4) What is the associated economic cost?

## Methods

Analysis of data from the 2009/2010 British Crime Survey. [8].

### Ethics Approval

In this study we analysed British Crime Survey (BCS) data collected on behalf of the Home Office, which is available to the academic community from the UK Data Archive (UKDA). A subset of this data (including data on domestic and sexual violence and on substance misuse) could only be accessed under Special License, to ensure participant confidentiality. We were granted Home Office approval to access BCS Special License data for the purposes of this study. We did not seek additional ethics committee approval for this secondary data analysis.

### Setting, Participants and Study Design

The BCS is a nationally representative cross-sectional survey of crime victimisation in men and women aged 16 or over living in private residential households in England and Wales. The survey has a complex design, with clustering, stratification and unequal sampling probability. [9] The sample size is powered to estimate crime rates in each of 42 Police Force Areas (PFAs), with a minimum target of 1000 participants per PFA. Lay interviewers collected data in door-to-door visits. The main survey used face-to-face interviews, and included measures of non-domestic and domestic physical and sexual violence. Sexual assaults and domestic violence were also measured using a more sensitive self-completion questionnaire, but only in those aged 16–59. This questionnaire was omitted if participants refused to complete it or asked for interviewer help. Historically, around 80% of BCS participants completed this questionnaire, with non-completers being older and more socially deprived. [10] Also historically, less than a fifth of those who reported sexual or domestic violence in the self-completion questionnaire also reported these experiences in the face-to-face interview. [10].

We included participants who took part in the April/2009- March/2010 BCS survey (when disability subtypes were first measured). We excluded individuals with missing data on disability or on survey design. We performed two sets of analyses: (1) An analysis of data on all participants, using violence measures from face-to-face interviews only (‘main-interview analyses’) (2) An analysis of data on the subgroup of people aged 16–59 who answered the self-completion questionnaire, using both face-to-face and self-completion violence measures (‘self-completer analyses’). Therefore, the former included all participants across the age range, whilst the latter included a younger subgroup with more sensitive measures of sexual and domestic violence.

### Measures (see Box1)

Disability was defined as any ‘long-standing physical or mental health conditions or disabilities that have lasted or are expected to last 12 months or more and which limit day to day activities’. Our main exposure was a three-level disability measure: (a) no disability (b) one or more disabilities, including disability due to mental illness (‘mental illness’) (c) one or more disabilities, excluding disability due to mental illness (‘non-mental disability’). The main outcome was being the victim of any actual or threatened violence in the past year (whether physical or sexual, domestic or non-domestic). Secondary outcomes were the following six violence subtypes: actual, threatened, physical, sexual, domestic and non-domestic violence. We adjusted for age, sex, social deprivation (at the individual, household and area levels) and substance misuse (see Box 1). [11] [2] Potential interaction terms were disability interacting with sex and age.

### Statistical Analysis

We carried out design-based analyses (which took into account the complex survey design, including weighting, clustering and stratification) using the ‘svy’ suite commands in Stata, version 11.0 (Stata Corporation, East College Station, TX USA). We report weighted prevalence estimates with robust standard errors. Hypothesis tests were based on adjusted Pearson’s tests (for bivariate analyses), or adjusted Wald tests (for multivariate logistic regression analyses).

We estimated the crude prevalence and age/sex adjusted odds ratios for any violence victimisation for each of the six disability subtypes (compared to those without the given disability). To address our first question, we estimated crude and age/sex standardised prevalence of violence and its subtypes in those with no disability, mental illness and non-mental disability; using the whole study sample as the standard population. We estimated odds ratios (ORs) for violence and its subtypes (a) adjusted for age and sex (b) adjusted for the other covariates detailed in Box 1. We tested the final models for interaction between disability and sex and disability and age using the interaction effect Wald test. To address our second question, we estimated the prevalence and odds of physical and mental ill health following violent offences experienced by those with and without disability (adjusting for age, sex and offence type; and for clustering of offences within individuals).

To address our third question, we used Greenland’s methodology [12], [13] to estimate the proportion of violence that can be attributed to the independent effect of disability (the population attributable fraction or PAF), both in the general population and among people with disability. Greenland’s methodology is recommended for estimating adjusted attributable risk (where the effect of other factors is taken into account). [13] It employs a maximum likelihood approach based on the logistic model. We used the Greenland-based ‘punaf’ command in Stata (V12.0 SE), which estimates PAFs on the basis of parameter estimates from multivariate logistic regression models.

To estimate the burden of disability-related violence at the population level, we combined 2009 Office for National Statistics population figures [14] with our estimates of disability prevalence, violence prevalence, and PAFs to estimate (a) the total number of people with disability who experienced violence (calculated as population total × prevalence of disability × prevalence of violence amongst those with disability; summed across 5 age-group strata to improve precision of estimates (b) the total number of people who experienced violence attributable to the independent effect of disability (calculated as (a) × PAF amongst people with disability).

Finally, we estimated the financial cost of violence attributable to disability in England & Wales (E&W) in 2009. We used the best available estimates of the unit costs of crime; which were first developed by the Home Office research directorate in 2000 [15], and most recently updated in 2011 [16]These unit costs are derived from estimates in the general population, and include costs to healthcare and criminal justice systems, lost economic output, and cost to victims from the physical and emotional impact of crime. [17] For violent crime, incidents are grouped into 5 cost categories (serious wounding, other wounding, common assault, robbery and sexual assaults). In the BCS, participants who report being a victim of violence are asked detailed information about the number of incidents experienced and the nature of each incident (e.g. who it was perpetrated by, injuries sustained, etc…). Each incident is then coded into one of the above categories. This data is provided by the Home Office alongside population weights, and hence allows for estimation of the total number of incidents experienced in the population. We estimated (a) the total cost of crime among those with disability (calculated as number of incidents experienced by people with disability in the study sample × population weights for these incidents × unit costs) and (b) the cost attributable to the effect of disability (calculated as (a) × our PAF estimate for main-module actual violence). These cost estimates did not include violence disclosed in the self-completion module, since there are no available up to date unit cost estimates for these experiences. [18], [19] This follows the methodology used by the Home Office, where the published cost of crime only includes violence disclosed in the main module. [17].

We examined the frequency of missing data for all covariates included in the model. For variables with missing data in more than 5% of participants, we included a ‘value missing’ category in logistic regression analyses. To assess for participation bias in the self-completion measures, we compared the characteristics of those who completed this module with those who were unwilling or unable to do so.

We carried out additional analyses to separate out the effects of disability type, number of co-morbid disabilities and severity of functional limitation on violence risk (see supplementary material).

## Results

### Participant Flow and Response Rates

In 2009/10, 44638 people participated in the BCS, a response rate of 76% (with significantly lower participation in those aged under 35 and over 60, in men and in London). [9] 44398/44638 (99.5%) of BCS respondents were included in our analysis, after excluding those who had missing survey design data (n = 149), or disability data (n = 91). 28225/44398 (64%) were aged under 60, and hence eligible for the self-completion questionnaire. Of those, 22874/28225 (81%) completed that questionnaire, with significantly lower participation in older people, men, ethnic minorities, the socially deprived and those with disability (69% vs. 82% of those with and without disability respectively; p<0.001).

### Socio-demographics and Prevalence of Disability

Sample socio-demographic characteristics are shown in table 1, and largely reflected the general population. 9037/44398 participants (16.2%) had at least one limiting disability; 7781 (13.9%) had one or more disabilities excluding mental illness (‘non-mental disability’) and 1256 (2.4%) had one or more disabilities including mental illness (‘mental illness’). Those with and without disabilities differed on most socio-demographic characteristics, with disabled people being significantly older (mean age 61 vs. 44, p<0.001), and more likely to be female and socially deprived (Table 1). Only household income had missing values for more than 1% of the sample (with missing values for 18% of the sample).

**Table 1:** Sample socio-demographic and disability characteristics.

	Non-disabled (N = 35361) % (n)	Disabled (N = 9037) % (n)
**Socio-demographic characteristics** *		
Mean age (sd)**	43.6 (sd 0.44)	61.0 (sd 0.52)
Female**	50.3 (19187)	56.0 (5225)
White**	88.1 (32498)	92.7 (8604)
Married/cohabiting**	63.7 (20914)	56.2 (3921)
Living alone**	11.7 (8054)	29.0 (4132)
Has degree/diploma**	36.6 (12670)	18.9 (1697)
Employed**	66.9 (22394)	20.9 (1591)
Renting social housing**	12.0 (4510)	30.4 (2908)
Living in urban area	79.0 (26051)	79.3 (6800)
Living in an inner city**	10.0 (2665)	12.0 (868)
Living in area in lowest deprivation quintile**	18.2 (5986)	28.2 (2454)
**Disability characteristics**	Not applicable	
Mobility impairment		52.0 (4930)
Sensory impairment		14.4 (1392)
Long-term physical illness		9.3 (815)
Learning disability		2.7 (170)
Mental health condition		14.5 (1256)
Other		51.0 (4563)
*(other only)*		*26.0 (2236)*
One or more disability excluding mental illness		85.5 (7781)
One or more disability including mental illness		14.5 (1256)
Severe functional disability		28.4 (2692)
Two or more disabilities		34.5 (3202)

* None of these variables had missing values for >1% of the sample.

** p for difference <0.001.

### Prevalence and Odds of Violence by Disability

Prevalence and odds of any violence for each of the disability subtypes are shown in Figure S1 and Table S1From the main interview analyses, the age/sex adjusted relative odds for victimisation were highest amongst those with mental illness (aOR 2.7; CI 2.2–3.4) and long-term physical illness (aOR 2.6, CI (1.8–3.7), followed by those with mobility problems (aOR 1.9, CI (1.6–2.3) and ‘other’ disability (aOR 2.0, CI 1.7–2.4). There was no association between violence victimisation and either sensory impairment (aOR 1.3, CI 0.9–2.0) or learning disability (aOR 0.8, CI 0.4–1.5) at the 5% significance level. We found similar associations in the self-completer analyses (see Figure S1 and Table S1).

**Figure 1:**
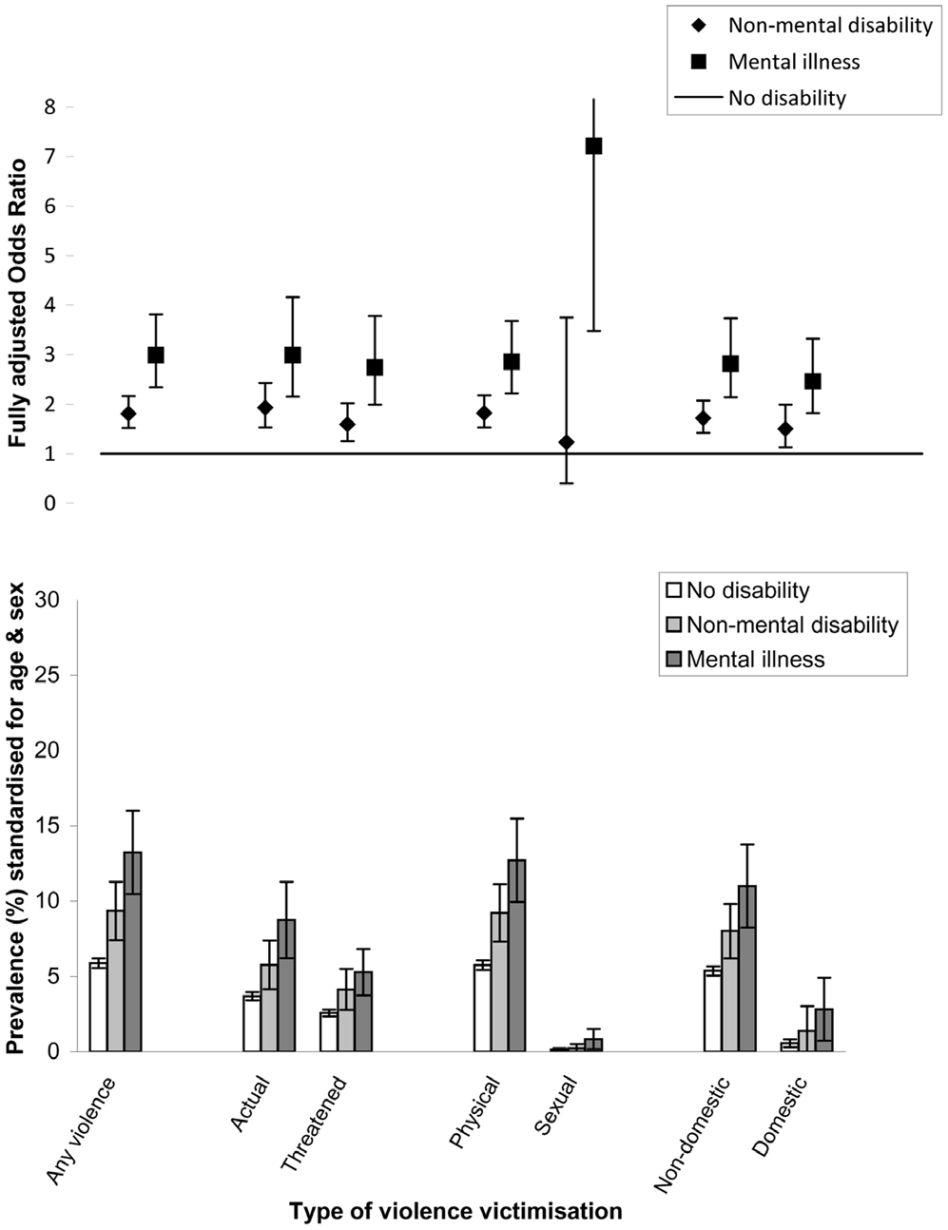
Prevalence and odds of violence subtypes in people aged 16 and above, by disability (interview measures of violence only).

**Figure 2:**
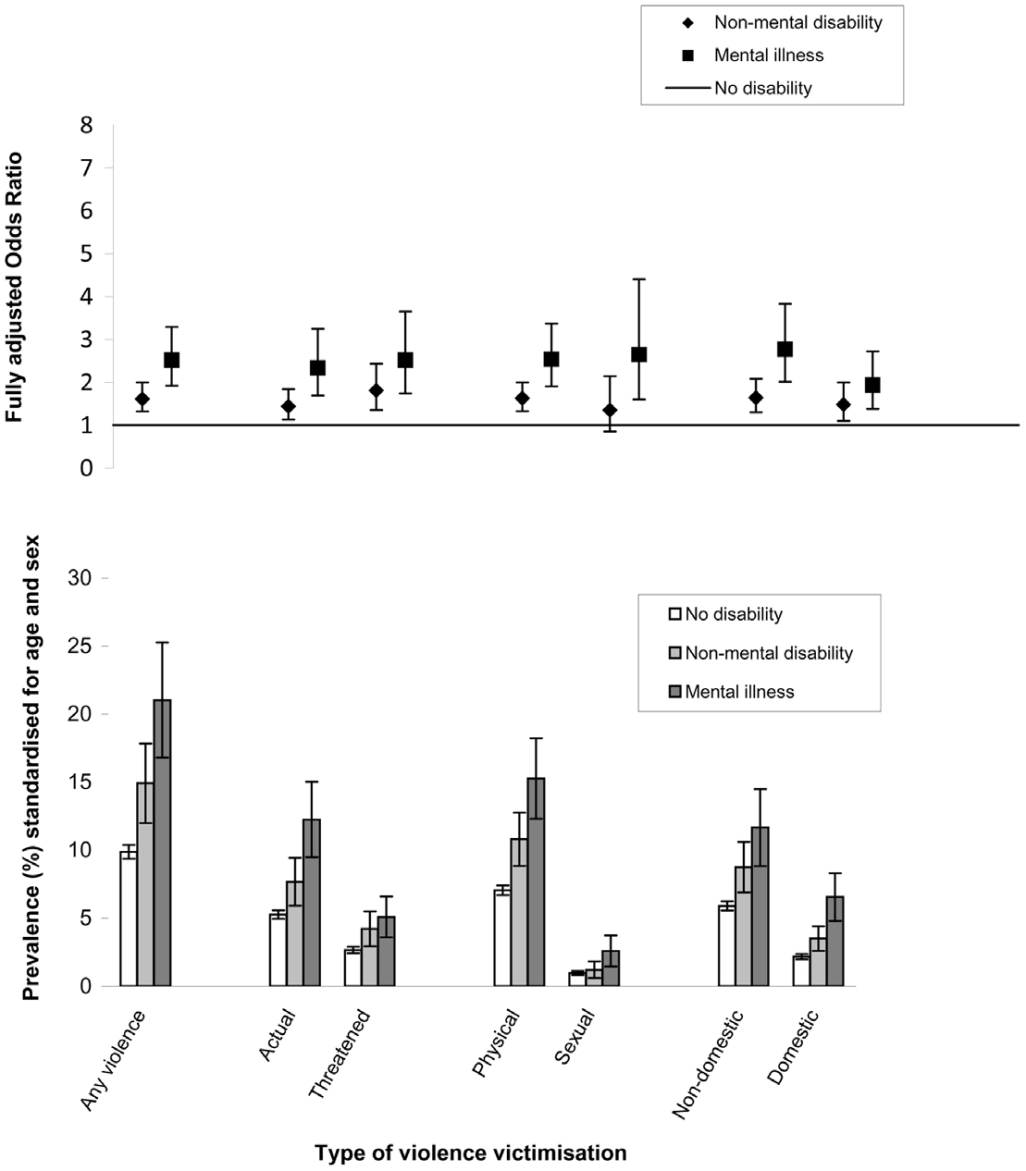
Prevalence and odds of violence subtypes in people aged 16–59, by disability (interview and self-completion measures of violence).

Prevalence and odds of violence and its subtypes for those with no disability, non-mental disability or mental illness are shown in Figures 1 & 2 and Tables S2 & S3. Age and sex standardised prevalence of any past-year actual or threatened violence in those with no disability, non-mental disability and mental illness was 5.9, 9.3 and 13.2% respectively in the main interview analyses, and 9.9, 14.9 and 21.0% respectively in the self-completer analyses. A similar gradient was observed across all violence subtypes. For those with self-completion data who reported victimisation, a similar proportion of victims with and without disability reported physical violence (92% and 91% respectively) and sexual violence (15% and 14% respectively). Although both victims with and without disability reported non-domestic violence more often than domestic violence, domestic violence was reported by a greater proportion of disabled than non-disabled victims (44% vs. 31%; p for difference<0.01), whilst non-domestic violence was reported by a lower proportion of disabled than non-disabled victims (66% vs. 74%; p for difference<0.001).

For all violence subtypes, and in both sets of analyses, the age/sex adjusted OR was higher in those with disability compared to those without (at the 1% significance level), and higher in those with mental illness than those with non-mental disability (at the 5% significance level). Across violence subtypes, those with non-mental disability had nearly double the odds and those with mental illness had nearly triple the odds of violence compared with the non-disabled after adjusting for age and sex. Additional adjustment for a range of individual, household and area factors resulted in only minimal changes to the OR estimates, except for a sizeable reduction in the OR of domestic violence-particularly among those with mental illness.

There was no interaction between disability and age or sex on violence risk. Regardless of disability, men were more likely to be victims of physical and non-domestic violence (53 & 58% of victims respectively; p<0.05), whilst women were much more likely to be victims of sexual and domestic violence (83 & 71% of victims respectively; p<0.001). Across all violence types, around 80% of the violence was perpetrated by men, 10% by women and 10% by both men and women.

Only 15% of those who reported sexual violence and 25% of those who reported domestic violence in the self-completion questionnaire also reported these experienced in the main interview, with no differences in disclosure rates by disability.

### Health Impact of Violent Incidents by Disability

Health impact was reported for a total of 2477 violent incidents, which were experienced by 2100 people who reported violence in the main module questionnaire (table 2). There were no differences between those with and without disability in the mean number of incidents experienced (1.5), or in the proportion of incidents resulting in physical injury (28.0%) or requiring medical attention (10.3%). Those with disability were more likely to report that the incident led to anxiety, depression or panic attacks, and that they were emotionally affected ‘very much or quite a lot’ rather than ‘just a little’ by the incident (at the 1% significance level). These adverse mental health effects were commoner in those with pre-existing mental illness than those with other disability types (at the 5% significance level).

**Table 2:** Impact of violent offences on heath, by disability.

	n/N of incidents^1^	%	OR (CI) adjusted for age, sex and offence type
**Injury**			
Non-disabled	531/1951	28.4	
Non-mental disability	78/330	23.2	
Mental illness	72/196	32.0	
Total	681/2477	28.0	
*p for difference*		*0.15*	
**Medical attention**			
Non-disabled	173/1951	10.3	
Non-mental disability	34/330	10.5	
Mental illness	25/196	9.6	
Total	232/2477	10.3	
*p for difference*		*0.96*	
**Emotionally affected 'quite a lot' or 'very much'**			
Non-disabled	425/1878	19.8	1
Non-mental disability	111/319	32.6	1.8 (1.3–2.5)
Mental illness	90/188	44.5	2.5 (1.6–3.9)
Total	626/2385	22.4	
*p for difference*		*<0.001*	
**Anxiety, depression or panic attacks**			
Non-disabled	240/1879	10.6	1
Non-mental disability	63/319	16.3	1.5 (0.95–2.2)
Mental illness	92/188	42.2	4.9 (3.2–7.6)
Total	395/2386	12.8	
*p for difference*		*<0.001*	

1 These incidents were experienced by 1653 people without disability, 290 people with non-mental disability and 157 people with mental illness.

### Population Attributable Fraction and Population Estimates

PAFs and related population estimates are shown in table 3. Using ‘main interview’ findings, which relate to people aged 16 and above, we estimated that the proportion of violence which could be attributed to the independent effect of disability was 7.5% (CI 5.7–9.3%) in the general population and 48.8% (CI 41.1–55.4%) among those with disability. Using ONS mid-2009 population figures for England and Wales we estimated that in that year the independent effect of disability resulted in an estimated additional 184,000 people with disability experiencing any actual or threatened violence, including 116,000 disabled victims of actual violence, at an excess cost of £1.51 billion pounds (table S4).

**Table 3:** Population attributable fraction for violence related to the independent effect of disability, and estimated number of victims arising from PAF in England and Wales in 2009^1^.

	PAF in whole population	PAF in those with disability	n all victims/N whole population (millions)^2^	n disabled victims/N disabled population (millions)^3^	n disabled victims attributable to disability (thousands)^4^
**Main interview analyses**					
Any violence	7.5 (5.7–9.3)	48.8 (41.1–55.4)	2.44/44.55	0.378/7.22	184.0
Actual violence	7.8 (5.4–10.1)	51.8 (41.5–60.4)	1.49/44.55	0.224/7.22	115.9
**Self-completer analyses**					
Any violence	4.8 (3.3–6.3)	41.2 (32.4–48.9)	3.22/32.26	0.375/2.66	154.9
Actual violence	6.2 (4.2–8.1)	43.1 (33.3–51.4)	2.17/32.26	0.236/2.66	134.2

1 Based on ONS mid-2009 population figures.

2 Based on (1) and our estimates of violence prevalence in the whole population.

3 Based on (1) and our estimates of prevalence of disability and prevalence of violence among the disabled.

4 Based on (3) and our PAF estimates among those with disability.

Estimates from the subgroup of people with self-completion data, which only relate to people aged 16–59, are summarised in Table S3. Compared to ‘main interview’ estimates, the PAFs are lower (reflecting the lower prevalence of disability in this younger subgroup), but the estimated number of victims are higher (reflecting the higher prevalence of violence when both self-completion and main interview measures are taken into account).

### Additional Analyses

Results from our additional analyses suggested that differences between the groups we defined as having disability with and without mental illness were due to the effect of mental illness itself, rather than to the differences between these groups in the number of co-morbid disabilities or the severity of functional limitation (see File S1).

## Discussion

Using a large general population sample, we found that people aged 16 and over with one or more disabilities including mental illness had relative odds of 3.0 (2.3–3.8) and those with one or more disabilities excluding mental illness had relative odds of 1.8 (1.5–2.2) of being a victim of past-year violence compared with the non-disabled after adjusting for socio-demographic and behavioural factors (with similar relative odds across violence subtypes). Compared with non-disabled victims, victims with disability were more likely to experience mental health problems following violent incidents, especially those with pre-existing mental illness. We estimated that around 8% of violence in the general population and half of violence among those with disability could be attributed to the independent effects of disability, and that this resulted in an estimated additional 116,000 people with disability experiencing actual violence in England and Wales in 2009, at an excess cost of £1.51 billion.

Overall, prevalence and risk estimates are consistent with studies from other countries. [5] In the USA, one national and one statewide household survey found that women with disability had four-fold the odds of being a victim of sexual assault than non-disabled women. [20], [21] Both studies found no association between disability and physical assaults, but this may be due to limited study power. In our much larger study, we found a clear association between disability and both physical and sexual assaults. In Taiwan, national data on sexual assaults found that people with disability were more likely to experience sexual assaults than those without, particularly those with learning difficulty and chronic psychosis. [22] This is consistent with our finding of high risk among those with mental illness. However, we failed to find an association between violence and either learning disability or sensory impairment. This may be due to limited power (only 170 people with learning disability participated in this study). It could also be due to participation bias; the survey was designed for the general population, and people with significant intellectual impairment or communication problems may have found it difficult to participate in the lengthy and detailed study interview.

We found that the relative odds of violence outside and within the home were equally high, with the former being more prevalent. However, the prevalence of domestic violence may have been underestimated due to response or disclosure bias. Disabled victims were less likely to complete the sensitive self-reported measure of domestic violence than non-disabled victims, and it is possible that non-completers were at higher risk. Disclosure of domestic violence may be particularly difficult for disabled victims, as they may be dependent on perpetrators, and fear increased violence or independence loss and institutionalization following disclosure. [23] Nonetheless, this study suggests that interventions for both non-domestic and domestic violence are required in this population. We found that social deprivation and substance misuse did not account for the excess risk of non-domestic violence, but did account for some of the excess domestic violence risk (especially amongst those with mental illness), suggesting that these factors could be appropriate intervention targets for addressing domestic violence risk.

Past evidence suggested that those with mental illness were at particularly high risk, but this was largely based on comparisons between studies with widely differing settings and measures. [5] Our study is one of the few to directly compare risks for those with self-defined mental illness versus other disability types in a community sample. We found that those with mental illness had significantly higher risks of violence victimisation, and were more likely to suffer mental ill health following violence, than those with other disabilities. This may be explained by a high concentration of intersecting risk factors at the personal, interpersonal, community and societal levels among those with mental illness [2] [24] These include high rates of exposure to childhood violence (e.g. parental domestic violence and childhood abuse), which predisposes to mental illness and personality difficulties, which in turn put people at risk of low self-esteem, interpersonal conflict, substance misuse and violence perpetration. [25] This constellation of problems increases the risk of victimization, and decreases the likelihood of exiting a cycle of violence. Our findings suggest that those with mental illness would be a suitable group for targeted intervention. The factors that put them at risk may start early and require broad and complex interventions. Future research should identify which subgroups of people with mental disorder are at greatest risk of victimization. One cohort study found that people with both common mental disorders (anxiety, depression) and severe mental illness (schizophrenia) were at increased risk of physical and sexual assaults. [26] Among people with severe mental illness, risk is highest for those with a history of childhood abuse, co-morbid substance misuse and social disadvantage (such as homelessness and poor social support) [27], [28], [29], [30], [31].

We estimated population attributable fractions (PAFs), as this “provides a bridge by which results of epidemiologic studies can be made relevant to public health policy”. [32] By estimating this measure, we would classically be making the assumption that disability ‘causes’ violence, and that there are interventions that can eliminate disability and the violence risks associated with it. [32] We acknowledge that the causal pathway between exposure to disability and violent victimization is poorly understood and is likely to involve a complex interplay of variables relating to victim, perpetrator and environment. However, in our analyses, we sought to estimate the proportion of violence attributable to disability that is not explained by factors shared with the general population (and measured in our study). Factors unique to those with disability may include decreased ability to understand danger, to escape from a perpetrator on whom they are dependent and to communicate experiences to health and legal authorities. [33]–[34] Whilst disability-related risk accounted for a relatively small proportion of violence in the general population, the estimated number of victims with disability arising from this excess risk, and associated economic costs, are sizeable. Although we used the best available costing measures from the general population, these may not account for differences in demography, baseline health and response to violence in the disabled. A significant proportion of violent crime cost arises from its physical and emotional impact on victims. [17] As we showed in this study, the psychological impact is greater among people with disability, so we are likely to have underestimated the true cost.

Strengths of this study include the large, nationally representative sample with detailed measures of disability, violence and covariates, which allowed us to generate robust estimates of violence prevalence, risk and population impact. The study has several limitations. The target population only included people living in private residential households, so findings cannot be generalized to people with disability living in residential or supported accommodation. Findings cannot be generalized to those who have significant communication or cognitive problems (of a severity that would preclude their participation in the BCS). The survey did not use a sensitive measure of sexual and domestic violence in those aged 60 and above, hence underestimating these violence subtypes in this age group. Although it is difficult to establish temporal relationships in cross-sectional surveys, those with disability had a minimum illness duration of 1 year, and the main outcome was past-year violence, so-apart from measurement errors- disability would have preceded violence. Reporting bias is possible, but its likely direction is unclear. People with mental illness or other disabilities may over-report violence since it has a greater impact on them. Conversely, they may under-report violence as they may worry more about the consequences of disclosure. Past evidence suggests that people with mental illness tend to reliably report victimisation experiences. [35].

Our research highlights the need for clinicians to be aware of the elevated risks of domestic and non-domestic violence among patients with all disability types; and of the increased risk of mental health problems among disabled victims. Although people with mental illness are the most vulnerable to violent victimisation, mental health professionals often fail to screen for recent abuse, and violence is rarely detected or acted upon. [36] A recent review on domestic violence interventions for people with disability found that disabled victims had difficulty accessing generic services. Specialist services were rarely available and had a poor evidence base– although there were some promising approaches, including safety training and peer support. [34] In the non-disabled population, there is good evidence that there are effective interventions for both primary violence prevention (e.g. parent training, life skills training for children and adolescents) and secondary violence prevention (e.g. screening tools, education programs for health professionals, advocacy support programs). [37]–[39] Future research should evaluate the effectiveness of these interventions among people with disability,; as well developing interventions to address risk factors specific to this group (e.g. caregiver stress, communication barriers to disclosure). From a policy perspective, our findings strengthen the economic and public health arguments for interventions in this group, and suggest the need for greater integration between health and victim support services.

People with disability, a predominantly elderly and disadvantaged group, are at increased risk of violence both within and outside the home. The significant public health and economic burden calls for an urgent assessment of the causes of this violence, and national policies on violence prevention in this vulnerable group.

## Supporting Information

Figure S1 **Adjusted odds of any violence victimisation, by disability subtype.**
(DOC)

Table S1 **Prevalence and odds of any violence victimisation, by disability subtype.**
(DOCX)

Table S2 **Prevalence and odds of violence victimisation subtypes in people aged 16 and over, by disability (‘main-interview analyses’).**
(DOCX)

Table S3 **Prevalence and odds of violence victimisation subtypes in people aged 16–59, by disability (‘self-completer analyses’).**
(DOCX)

Table S4 **Estimates of number of victimisation incidents and associated cost, by disability.**
(DOCX)

File S1 **Supplementary Information-additional analyses.**
(DOCX)
