# Supplementary material for: Violence against People with Disability in England and Wales: Findings from a National Cross-Sectional Survey
Source: PLoS One. 2013 Feb 20;8(2):e55952. doi: 10.1371/journal.pone.0055952 (PMC3577814; doi:10.1371/journal.pone.0055952)
Supplement: Figure S1 — Adjusted odds of any violence victimisation, by disability subtype. (DOC) [file pone.0055952.s001.doc]

**Figure S1. Adjusted odds of any violence victimisation, by disability subtype**
