# Supplementary material for: Violence against People with Disability in England and Wales: Findings from a National Cross-Sectional Survey
Source: PLoS One. 2013 Feb 20;8(2):e55952. doi: 10.1371/journal.pone.0055952 (PMC3577814; doi:10.1371/journal.pone.0055952)
Supplement: Table S1 — Prevalence and odds of any violence victimisation, by disability subtype. (DOCX) [file pone.0055952.s002.docx]

Table S1 Prevalence and odds of any violence victimisation, by disability subtype

| Disability type | Main interview analyses | | Self-completer analyses | |  |
| --- | --- | --- | --- | --- | --- |
|  | Violence prevalence^1^ (n/N) | Age / sex adjusted OR (95% CI)^2^ | *% with SC data of those eligible for SC module (n/N)* | Violence prevalence^1^ (n/N) | Age / sex adjusted OR (95% CI)^2^ |
|  |  |  |  |  |  |
| No disability | 5.53 (1653/35361) | - | *82 (20585/24941)* | 9.6 (1868/20585) | - |
| Any disability | 5.23 (447/9037) | 2.05 (1.8-2.3) | *69 (2289/3314)* | 14.1 (352/2289) | 2.0 (1.7-2.4) |
|  |  |  |  |  |  |
| Mental Illness | 11.6 (157/1256) | 2.7 (2.2-3.4) | *71 (676/959)* | 20.1 (148/676) | 2.7 (2.1-3.5) |
| Long-term illness | 7.3 (50/815) | 2.6 (1.8-3.7) | *68 (232/342)* | 18.8 (43/232) | 2.8 (1.9-4.3) |
| Mobility problems | 3.8 (184/4930) | 1.9 (1.6-2.3) | *67 (840/1250)* | 12.9 (122/840) | 2.0 (1.6-2.5) |
| Sensory impairment | 2.9 (38/1392) | 1.3 (0.89-2.0) | *60 (171/283)* | 11.4 (26/171) | 1.4 (0.9-2.5) |
| Learning disability | 6.1 (12/170) | 0.77 (0.39-1.5) | *39 (55/141)* | 11.7 (11/55) | 0.96 (0.46-2.0) |
| ‘Other’ | 5.5 (235/4563) | 2.0 (1.7-2.4) | *68 (1166/1713)* | 13.0 (163/1166) | 1.8 (1.5-2.3) |

1. Crude prevalence, taking into account survey weights and clustering

2. The baseline group was people without the given disability
