# Supplementary material for: Violence against People with Disability in England and Wales: Findings from a National Cross-Sectional Survey
Source: PLoS One. 2013 Feb 20;8(2):e55952. doi: 10.1371/journal.pone.0055952 (PMC3577814; doi:10.1371/journal.pone.0055952)
Supplement: Table S2 — Prevalence and odds of violence victimisation subtypes in people aged 16 and over, by disability (‘main-interview analyses’). (DOCX) [file pone.0055952.s003.docx]

Table S2 Prevalence and odds of violence victimisation subtypes in people aged 16 and over, by disability (based on interview measures of violence only; ‘main-interview analyses’)

|  | **n/N** | | **% of victims experiencing violence subtype** | **Crude prevalence** | **Age/sex standardised prevalence (CI)** | **OR adjusted for age & sex (CI) ^1^** | **Fully adjusted OR (CI) ^1, 2^** |
| --- | --- | --- | --- | --- | --- | --- | --- |
| **Any violence** | |  |  |  |  |  |  |
| No disability | | 1653/35361 | 100 | 5.5 (5.2-5.9) | 5.9 (5.6-6.2) | 1 | 1 |
| Non-mental disability | | 290/7781 | 100 | 4.1 (3.6-4.7) | 9.3 (7.4-11.3) | 1.8 (1.5-2.1) | 1.8 (1.5-2.2) |
| Mental illness | | 157/1256 | 100 | 11.6 (9.7-14.0) | 13.2 (10.5-16.0) | 2.9 (2.3-3.7) | 3.0 (2.3-3.8) |
| *Total* | | *2100/44398* | *100* | *5.5 (5.2-5.8)* |  |  |  |
| **Actual violence** | |  |  |  |  |  |  |
| No disability | | 970/35361 | 62 | 3.4 (3.2-3.7) | 3.7 (3.4-4.0) | 1 | 1 |
| Non-mental disability | | 171/7781 | 57 | 2.4 (2.0-2.9) | 5.8 (4.2-7.4) | 1.9 (1.6-2.4) | 1.9 (1.5-2.4) |
| Mental illness | | 97/1256 | 60 | 7.0 (5.4-9.0) | 8.7 (6.2-11.3) | 3.1 (2.3-4.2) | 3.0 (2.2-4.2) |
| *Total* | | *1238/44398* | *61* | *3.4 (3.1-3.6)* |  |  |  |
| **Threats of violence** | |  |  |  |  |  |  |
| No disability | | 771/35361 | 45 | 2.5 (2.3-2.7) | 2.6 (2.4-2.8) | 1 | 1 |
| Non-mental disability | | 131/7781 | 45 | 1.9 (1.5-2.2) | 4.1 (2.8-5.5) | 1.5 (1.2-1.9) | 1.6 (1.3-2.0) |
| Mental illness | | 72/1256 | 45 | 5.2 (3.9-6.9) | 5.3 (3.7-6.8) | 2.4 (1.8-3.3) | 2.7 (2.0-3.8) |
| *Total* | | *974/44398* | *45* | *2.5 (2.3-2.7)* |  |  |  |
| **Physical violence** | |  |  |  |  |  |  |
| No disability | | 1617/35361 | 98 | 5.4 (5.1-5.7) | 5.7 (5.4-6.1) | 1 | 1 |
| Non-mental disability | | 286/7781 | 99 | 4.1 (3.6-4.7) | 9.2 (7.3-11.1) | 1.8 (1.5-2.1) | 1.8 (1.5-2.2) |
| Mental illness | | 147/1256 | 94 | 11.0 (9.0-13.3) | 12.7 (9.9-15.5) | 2.8 (2.2-3.6) | 2.9 (2.2-3.7) |
| *Total* | | *2050/44398* | *98* | *5.4 (5.1-5.6)* |  |  |  |
| **Sexual violence** | |  |  |  |  |  |  |
| No disability | | 43/35361 | 2.4 | 0.14 (0.09-0.20) | 0.16 (0.10-0.22) | 1 | 1 |
| Non-mental disability | | 5/7781 | 1.3 | 0.06 (0.02-0.14) | 0.22 (-0.06-0.51) | 1.2 (0.4-3.3) | 1.2 (0.4-3.8) |
| Mental illness | | 13/1256 | 8.0 | 0.93 (0.50-0.17) | 0.84 (0.17-1.52) | 7.5 (3.7-15.1) | 7.2 (3.5-15.0) |
| *Total* | | *61/44398* | *2.7* | *0.15 (0.11-0.20)* |  |  |  |
| **Stranger/acquaintance violence** | |  |  |  |  |  |  |
| No disability | | 1440/35361 | 92 | 5.0 (4.7-5.4) | 5.4 (5.0-5.7) | 1 | 1 |
| Non-mental disability | | 244/7781 | 85 | 3.5 (3.0-4.0) | 8.0 (6.2-9.8) | 1.6 (1.4-1.9) | 1.7 (1.4-2.1) |
| Mental illness | | 118/1256 | 81 | 9.4 (7.5-11.7) | 11.0 (8.2-13.8) | 2.6 (2.0-3.4) | 2.8 (2.1-3.7) |
| *Total* | | *1802/44398* | *90* | *4.9 (4.7-5.2)* |  |  |  |
| **Domestic violence** | |  |  |  |  |  |  |
| No disability | | 226/35361 | 9 | 0.52 (0.44-0.61) | 0.56 (0.47-0.65) | 1 | 1 |
| Non-mental disability | | 43/7781 | 14 | 0.60 (0.41-0.87) | 1.4 (0.73-2.0) | 2.7 (1.8-4.2) | 1.5 (1.1-2.0) |
| Mental illness | | 43/1256 | 22 | 2.60 (1.9-3.6) | 2.8 (1.8-3.9) | 5.2 (3.5-7.8) | 2.5 (1.8-3.3) |
| *Total* | | *312/44398* | *11* | *0.58 (0.51-0.66)* |  |  |  |

1. The OR were significantly higher for those with disability compared to those without (at the 1% sig. level) and for those with mental illness compared to those with non-mental disability (at the 5% sig. level) for all violence types.
2. OR adjusted for age, sex, ethnicity, marital status, individual and household social deprivation, substance misuse and area factors (see Box 1 for details)
