# Supplementary material for: Violence against People with Disability in England and Wales: Findings from a National Cross-Sectional Survey
Source: PLoS One. 2013 Feb 20;8(2):e55952. doi: 10.1371/journal.pone.0055952 (PMC3577814; doi:10.1371/journal.pone.0055952)
Supplement: Table S3 — Prevalence and odds of violence victimisation subtypes in people aged 16–59, by disability (‘self-completer analyses’). (DOCX) [file pone.0055952.s004.docx]

Table S3 Prevalence and odds of violence victimisation subtypes in people aged 16-59, by disability (based on interview and self-completion measures of violence; ‘self-completer analyses’)

|  | **n/N** | **% of victims experiencing violence subtype** | **Crude prevalence** | **Age/sex standardised prevalence (CI)** | **OR adjusted for age & sex (CI) ^1^** | **Fully adjusted OR (CI) ^1, 2^** |
| --- | --- | --- | --- | --- | --- | --- |
| **Any violence** |  |  |  |  |  |  |
| No disability | 1868/20585 | 100 | 9.6 (9.1-10.1) | 9.9 (9.4-10.4) | 1 | 1 |
| Non-mental disability | 204/1613 | 100 | 11.9 (10.1-13.8) | 14.9 (12.0-17.8) | 1.7 (1.4-2.1) | 1.6 (1.3-2.0) |
| Mental illness | 148/676 | 100 | 20.1 (16.7-23.9) | 21.0 (16.8-25.3) | 2.9 (2.2-3.7) | 2.5 (1.9-3.3) |
| *Total* | *2220/22874* | *100* | *10.0 (9.5-10.5)* |  |  |  |
| **Actual violence** |  |  |  |  |  |  |
| No disability | 1273/20585 | 68 | 6.6 (6.2-7.0) | 5.3 (5.0-5.6) | 1 | 1 |
| Non-mental disability | 133/1613 | 60 | 7.1 (5.8-8.6) | 7.7 (5.9-9.4) | 1.6 (1.3-2.0) | 1.4 (1.1-1.8) |
| Mental illness | 104/676 | 68 | 13.7 (10.8-17.3) | 12.3 (9.5-15.0) | 2.9 (2.2-3.9) | 2.3 (1.7-3.3) |
| *Total* | *1510/22874* | *68* | *6.7 (6.4-7.1)* |  |  |  |
| **Threats of violence** |  |  |  |  |  |  |
| No disability | 664/20585 | 35 | 3.3 (3.0-3.7) | 2.7 (2.4-2.9) | 1 | 1 |
| Non-mental disability | 79/1613 | 45 | 5.3 (4.1-6.7) | 4.2 (2.9-5.5) | 1.8 (1.4-2.4) | 1.8 (1.4-2.4) |
| Mental illness | 54/676 | 37 | 7.5 (5.5-10.1) | 5.1 (3.6-6.6) | 2.4 (1.7-3.4) | 2.5 (1.7-3.7) |
| *Total* | *797/22874* | *36* | *3.6 (3.2-3.9)* |  |  |  |
| **Physical violence** |  |  |  |  |  |  |
| No disability | 1701/20585 | 91 | 8.7 (8.2-9.2) | 7.1 (6.7-7.4) | 1 | 1 |
| Non-mental disability | 188/1613 | 92 | 10.9 (9.2-12.7) | 10.8 (8.9-12.8) | 1.7 (1.4-2.1) | 1.6 (1.3-2.0) |
| Mental illness | 133/676 | 92 | 18.3 (15.0-22.1) | 15.3 (12.3-18.2) | 2.9 (2.2-3.7) | 2.5 (1.9-3.4) |
| *Total* | *2022/22874* | *91* | *9.0 (8.6-9.5)* |  |  |  |
| **Sexual violence** |  |  |  |  |  |  |
| No disability | 243/20585 | 14 | 1.3 (1.1-1.5) | 1.0 (0.8-1.1) | 1 | 1 |
| Non-mental disability | 25/1613 | 12 | 1.4 (0.9-2.2) | 1.2 (0.6-1.8) | 1.5 (0.9-2.3) | 1.4 (0.9-2.1) |
| Mental illness | 32/676 | 19 | 3.7 (2.5-5.5) | 2.6 (1.5-3.7) | 3.1 (2.0-4.9) | 2.7 (1.6-4.4) |
| *Total* | *300/22874* | *14* | *1.4 (1.2-1.6)* |  |  |  |
| **Stranger/acquaintance violence** |  |  |  |  |  |  |
| No disability | 1242/20585 | 74 | 7.1 (6.6-7.6) | 5.9 (5.6-6.2) | 1 | 1 |
| Non-mental disability | 130/1613 | 66 | 7.8 (6.5-9.5) | 8.8 (6.9-10.6) | 1.6 (1.3-2.0) | 1.6 (1.3-2.1) |
| Mental illness | 88/676 | 65 | 13.1 (10.2-16.7) | 11.7 (8.8-14.5) | 2.6 (1.9-3.5) | 2.8 (2.0-3.8) |
| *Total* | *1460/22874* | *73* | *7.3 (6.9-7.7)* |  |  |  |
| **Domestic violence** |  |  |  |  |  |  |
| No disability | 714/20585 | 31 | 3.0 (2.7-3.3) | 2.2 (2.0-2.4) | 1 | 1 |
| Non-mental disability | 86/1613 | 39 | 4.6 (3.6-5.9) | 3.5 (2.6-4.4) | 1.9 (1.4-2.5) | 1.5 (1.1-2.0) |
| Mental illness | 74/676 | 43 | 8.6 (6.6-11.2) | 6.6 (4.8-8.3) | 3.1 (2.3-4.3) | 1.9 (1.4-2.7) |
| *Total* | *874/22874* | *32* | *3.2 (3.0-3.5)* |  |  |  |

1. The ORs were significantly higher for those with disability compared to those without (at the 1% sig. level) for all violence types. The OR were also significantly higher for those with mental illness compared to those with non-mental disability (at the 5% sig. level) for all violence types, expect for a non-significant difference in the fully adjusted OR for threatened violence (p=0.13) and for domestic violence (p=0.17).
2. OR adjusted for age, sex, ethnicity, marital status, individual and household social deprivation, substance misuse and area factors (see Box 1 for details)
