# Supplementary material for: Violence against People with Disability in England and Wales: Findings from a National Cross-Sectional Survey
Source: PLoS One. 2013 Feb 20;8(2):e55952. doi: 10.1371/journal.pone.0055952 (PMC3577814; doi:10.1371/journal.pone.0055952)
Supplement: Table S4 — Estimates of number of victimisation incidents and associated cost, by disability. (DOCX) [file pone.0055952.s005.docx]

Table S4. Estimates of number of victimisation incidents experienced and associated cost, by disability

|  | **Estimated N incidents in E&W in 2009 (1000s) ^1^** | | | **Estimated total cost in £billions (range)^2^** | | |
| --- | --- | --- | --- | --- | --- | --- |
| **INCIDENT TYPE** | Non-disabled | Non-mental disability | Mental illness | Non-disabled | Non-mental disability | Mental illness |
| Serious wounding | 91.5 (60.7-122.3) | 16.9 (2.6-31.3) | 11.5 (1.9-21.1) | 2.4 (1.6-3.1) | 0.4 (0.1-0.8) | 0.3 (0.0-0.5) |
| Other wounding | 336.8 (250.2-423.4) | 28.9 (8.1-49.8) | 26.1 (10.7-41.5) | 3.3 (2.4-4.1) | 0.3 (0.1-0.5) | 0.3 (0.1-0.4) |
| Common assault | 1128.1 (1003.2-1253.1) | 139.3 (99.1-179.5) | 60.7 (34.6-86.8) | 2.0 (1.8-2.2) | 0.2 (0.2-0.3) | 0.1 (0.1-0.2) |
| Robbery | 348.1 (271.1-425.1) | 41.5 (26.5-56.4) | 29.7 (11.9-47.5) | 3.1 (2.4-3.7) | 0.4 (0.2-0.5) | 0.3 (0.1-0.4) |
| Sexual offences | 70.1 (29.9-110.3) | 8.7 (-1.6-19.1) | 9.3 (0.8-17.7) | 2.6 (1.1-4.1) | 0.3 (-0.1-0.7) | 0.3 (0.0-0.7) |
| Total | 1974.7 (1615.2-2334.2) | 235.3 (134.7-336.0) | 137.3 (60.0-214.5) | 13.3 (9.3-17.3) | 1.7 (0.5-2.8) ^3^ | 1.3 (0.4-2.2) ^3^ |

1. BCS incident counts, weighted by population weights provided by the Home Office
2. Range based on uncertainty of incident count estimates. No confidence intervals were provided with the unit costs and could not be obtained by the authors, so cost uncertainty is not included
3. The estimated total cost in those with disability was £2.91 billion. The disability-related PAF for actual violence among those with disability was 51.8% (table 3). We therefore estimated the disability-associated excess risk as= £2.91 billion * 0.518=£1.51 billion
