# Supplementary material for: Violence against People with Disability in England and Wales: Findings from a National Cross-Sectional Survey
Source: PLoS One. 2013 Feb 20;8(2):e55952. doi: 10.1371/journal.pone.0055952 (PMC3577814; doi:10.1371/journal.pone.0055952)
Supplement: File S1 — Supplementary Information-additional analyses. (DOCX) [file pone.0055952.s006.docx]

**Supplementary material: additional analyses**

The BCS measured disability due to six chronic health conditions (see Box 1). , Nearly a third of participants had multiple disabilities (mean 1.4, range 1-6). A key study question was whether disability due to mental illness was associated with higher violence victimization risk than disability due to other health conditions. We therefore grouped those with disability into those who did or did not have mental illness. As there may have been differences between the two groups in the number of co-morbid chronic conditions or the extent of functional impairment, we carried out two additional analyses to check that it was mental illness per se rather than co-morbidity or extent of functional impairment which accounted for any excess risk. The first estimated the OR for violence in the whole sample for the following 5-level exposure variable: (a) no disability (b) one non-mental disability (c) two or more disabilities excluding mental illness (d) mental illness only (e) one or more disabilities including mental illness, adjusted for age and sex. The ORs for groups (b) to (e) were 1.7 (CI 1.4-2.0), 2.1 (CI 1.6-2.8), 2.6 (CI 1.8-3.6) and 3.4 (CI 2.4-4.5) respectively. The second analysis included the subgroup of people with disabilities, and estimated the independent effects of the following of the risk of any violent victimisation, adjusting for age and sex: (a) each of the six chronic conditions measured in the BCS(b) the number of co-morbid conditions (c) the severity of functional limitation. We found that only mental illness was independently positively associated with violence (OR 1.51, CI 1.02-2.25, p=0.04) . These findings suggest that differences between the groups we defined as having disability with and without mental illness were due to the effect of mental illness itself, rather than to the differences between these groups in the number of co-morbid disabilities or the severity of functional limitation.
